# GC-MS Analysis of Membrane-Graded Fulvic Acid and Its Activity on Promoting Wheat Seed Germination

**DOI:** 10.3390/molecules21101363

**Published:** 2016-10-13

**Authors:** Yi Qin, Hui Zhu, Mi Zhang, Huifen Zhang, Cheng Xiang, Baocai Li

**Affiliations:** 1 Faculty of Life Science and Technology, Kunming University of Science and Technology, Kunming 650500, China; qiny1985@163.com (Y.Q.); midylee@126.com (M.Z.); xcheng0871@163.com (C.X.); 2 School of Chemistry and Chemical Engineering, Sichuan University of Arts and Science, Dazhou 635000, China; zh326552917@163.com; 3 Food Security Research Institute of Yunnan Province, Kunming University of Science and Technology, Kunming 650500, China; 13085340138@163.com

**Keywords:** chemical component, fulvic acid, GC-MS, membrane-graded, seed germination

## Abstract

The chemical composition of fulvic acid (FA) with a molecular weight below 500 (FA-500) was analyzed, and its activity on promoting the seed germination of wheat was studied in this paper. The FA-500 was obtained by membrane separation technology and qualitatively and quantitatively analyzed by using gas chromatography-mass spectrometry combined with the retention index. Forty-seven constituents were identified, including structures with ester, acid and alcohol groups, which accounted for 95% of the total composition. The highest relative content of compounds was diethyl succinate and diethyl malonate, accounting for 29% and 17% of the total, respectively. Yannong 19 and Luyuan 301 wheat seeds were steeped with the FA-500 solution of different concentration respectively for two hours. Several markers were assessed: germination rate, coleoptile and radicle length, germination index, vitality index and the activity of α-amylase and (α+β) amylase. The results indicated that FA-500 had a significant effect on promoting seed germination within an appropriate concentration range. The best concentration was 0.5‰, and an inhibiting effect would appear with the increase of concentration. In the process of seed germination, FA-500 may affect the growth of the seed through influencing the amylase activity, which was related to respiration.

## 1. Introduction

Humic acid (HA) is a kind of amorphous macromolecular organic substance, which is mainly formed by a series of biological and geochemical process from plant remains [1]. HA exists widely in soil and waters and has higher content in weathered coal, peat and young lignite [2,3]. HA includes black humic acid, hymatomelanic acid and fulvic acid (FA) in accordance with the molecular weight and the differences of solubility in different solvents [3,4]. Compared with black humic acid and hymatomelanic acid, FA has a lower molecular weight, higher solubility, stronger biological activity, more active functional groups and is more easily absorbed by organisms [5]. Many research works show that FA has many plant physiological activities, such as promoting resistance to salt and drought and regulating plant growth [6,7,8]. Furthermore, FA possesses various pharmacological properties, such as anti-viral, anti-ulcer, inhibiting Alzheimer’s disease, adjusting immunity and sobering-up activities [9,10,11,12].

The physiological and chemical properties of HA or FA are often closely related to their molecular weight. HA of Iowa Soils was studied by Mao et al. [13] using infrared spectroscopy, nuclear magnetic resonance and mass spectrometry, and the results indicated that some characteristics of HA were related to the molecular size. HA molecules were divided into different molecular masses of eight grades by Li et al. [14,15] using ultrafiltration, and every grade was determined by high-performance size-exclusion chromatography, elemental analysis and nuclear magnetic resonance, of which the results showed that there were obvious differences on the structural characteristics and chemical properties among the eight grades of HA. In previous studies, we also found that a kind of FA substance with small molecules had a strong activity on promoting wheat seed germination at a very low concentration and meanwhile could alleviate salt injury in the wheat germination stage [16]. One of the reasons might be that small molecules were more easily absorbed by plants. Generally, the lower the molecular weight of FA, the greater the activity [17,18]. HA usually showed complexity of composition and a difference of function due to the multiplicity of raw material and different extraction processes, which created barriers for the research of the activity and its mechanism [19,20]. The molecular weight and chemical composition identification of humic substances, especially fulvic acids, is both the basis of and presents urgent needs for their activity studies. 

Accordingly, we gave priority to the research of the chemical composition of the low molecular weight fulvic acids. Considering that FA has a broad molecular weight distribution and complex structure, special analytical methods are required for characterization. In this study, the FA sample with a molecular weight less than 500 daltons (FA-500), obtained from membrane separation technology, was analyzed by using the method of GC-MS combined with the retention index (RI). Meanwhile, the activity on promoting seed germination of the small molecular fraction of fulvic acids was further explored. The small molecule fraction of fulvic acids usually has strong activities at extremely low concentrations, which is very suitable for the development of high efficiency organic fertilizer, such as foliar fertilizer, drip irrigation fertilizer and plant growth regulators. This implies potential large economic value. The component identification and activity investigation of FA-500 in this study may provide a theoretical basis and technical support for the development of the high-end HA fertilizer.

## 2. Results and Discussion

### 2.1. Chemical Composition Analysis of FA-500

**Figure 1:**
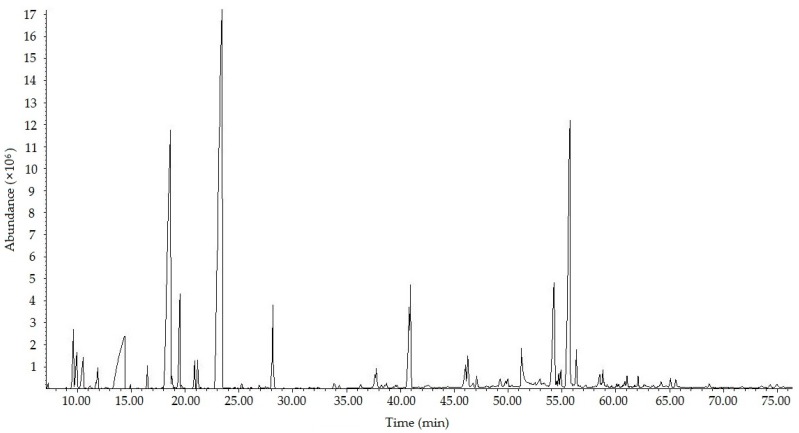
The total ion current chromatogram of fulvic acid with a molecular weight below 500 (FA-500).

A small amount of FA-500 solid was dissolved in ethanol (100 μg/mL); the total ion current chromatogram of FA-500 was obtained through the conditions mentioned below, as shown in Figure 1.

GC-MS was employed to separate and identify the chemical composition of FA-500 by combining with the RI values, and the relative content of the components was obtained through the method of peak area normalization. Forty-seven constituents from FA-500 with great similarity were acquired under the condition of the matching degrees of positive and negative. The name, retention time and the similarity of the components are shown in Table 1.

Table 1 showed that the relative content of all 47 constituents was more than 0.1%. These compounds accounted for 95% of the total composition. The highest mass fractions of the compounds were diethyl succinate and diethyl malonate, accounting for 29% and 17% of the total, respectively. The rest of the components with a higher content were ethyl hydrogen succinate, diethyl oxalate, ethyl hydrogen malonate and ethyl glycolate. The main ingredients were ethyl esters, which came up to 89%, and the rest was a small amount of acids and alcohols. Among them, the ethyl oxamate, DL-glutamine and diethyl 2-aminomalonate accounted for 0.231%, 0.233% and 0.277% respectively. These components are an indispensable nitrogen source for the plant [21,22]. The studies from Kelly et al. and Li et al. [23,24] showed that glutamine can promote the release of insulin from pancreatic cells and the generation of glutaminase plants to improve the activity of animals and plants. 

**Figure 2:**
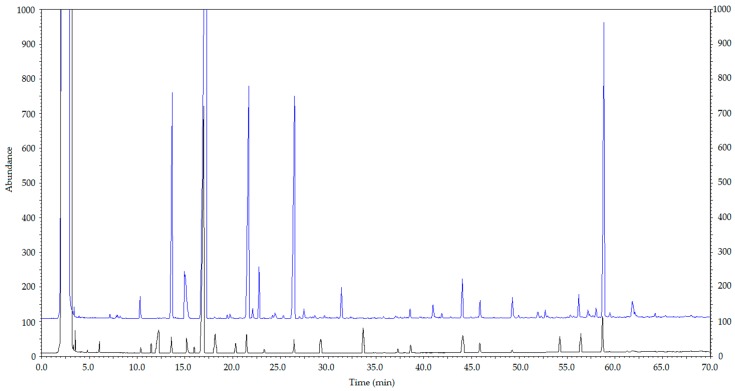
The gas chromatogram of 500-FA and the mixed standard sample. Note: the top is the gas chromatogram of 500–FA; the bottom is the gas chromatogram of the mixed standard sample.

To prove the validity of the results, the obtainable identified constituents had been mixed according to the relative content and dissolved with ethanol. Based on the conditions mentioned below, we obtained the spectra of 500-FA and the mixed standard sample, and the results are shown in Figure 2.

Figure 2 indicates that the material identified by the database had a great match with the standard mixture. The identified compounds of high content mostly had a good match with the mixed standard sample, but part of the material still was able to be matched. This implied that the result of the identification was erroneous or that there was the presence of isomers.

In this experiment, ethanol was chosen as the solvent. Other common solvents, such as acetone, acetonitrile, n-hexane, cyclohexane and ethyl acetate, cannot easily dissolve the sample and mixture. The pH of the 1% FA-500 solution was 2.1, while the main components identified were ethyl esters. It was supposed that there was a reaction between the acids and the solvent. This suggested that the choice of non-interfering solvent may be an important research direction of GC-MS analysis for FA. 

### 2.2. Influence of FA-500 on Germination Rate, Coleoptile and Radicle Length of Wheat Seed

Table 2 revealed that FA-500 had an influence on the germination rate and length of coleoptile and radicle under the tested concentration. When the concentration gradient of FA-500 was 0.3‰, 0.5‰ and 0.7‰, the values of the germination rate and the length of coleoptile and radicle were higher than the control group for both kinds of seeds. At the concentration of 0.5‰, the activity of FA-500 was the most obvious, which produced a significant effect on the length of Luyuan 301 coleoptile and radicle and the length of Yannong 19 radicle (*p* < 0.05). Meanwhile, the germination rate, the length of coleoptile and radicle for Yannong 19 increased by 1.6%, 9.5% and 16.1%, respectively. The three indexes of Luyuan 301 increased by 1.4%, 7.2% and 16.9%, respectively. However, the activity data of Yannong 19 and Luyuan 301 are similar. When the concentration was higher than 0.5‰, the three indexes of both kinds of seed began to be inhibited to varying degrees.

### 2.3. Influence of FA-500 on the Germination Index and the Vitality Index of Wheat Seed

**Figure 3:**
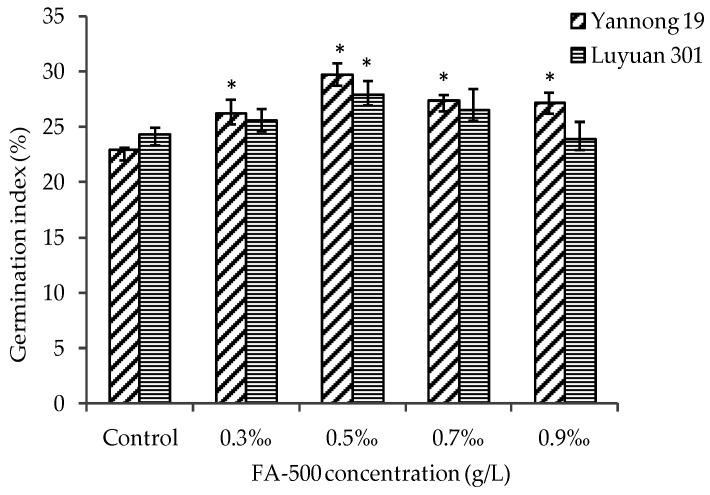
The effects of FA-500 with different concentrations on the germination index of wheat seed. Results are expressed as the mean ± S.D. (* *p* < 0.05 vs. control).

**Figure 4:**
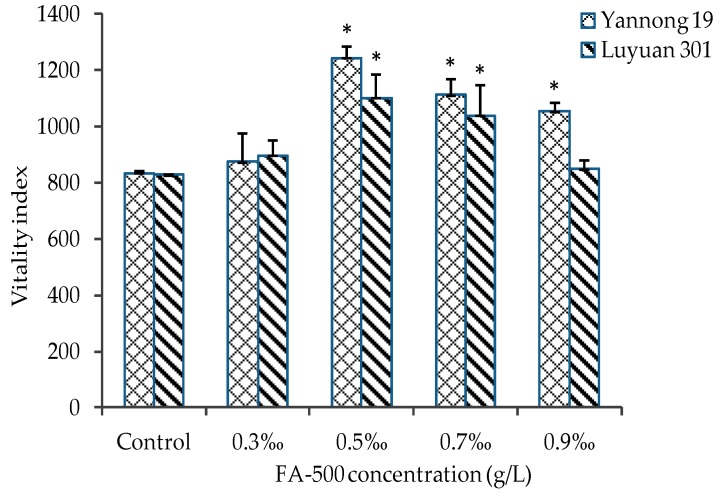
The effects of FA-500 with different concentrations on the vitality index of wheat seed. Data are expressed as the mean ± S.D. (* *p* < 0.05 vs. control).

Compared with the blank control group, the germination index and vitality index of both kinds of seed were improved significantly (*p* < 0.05) under most concentrations of FA-500 (Figure 3 and Figure 4). Overall, the two indexes of Yannong 19 were higher than Luyuan 301, which reflected that FA-500 had more significant activity on promoting germination for Yannong 19. At the concentration of 0.5‰, both indexes were the best. The two indexes of Yannong 19 increased by 29.7% and 49.1%, respectively, and 14.8% and 32.9% separately for Luyuan 301. When the concentration was higher than 0.5‰, there was a downward trend with the increase of concentration. When the concentration reached 0.9‰, the gap between the two kinds of seed was the largest for both indexes. 

### 2.4. Influence of FA-500 on the Activity of α-Amylase and (α+β) Amylase

**Figure 5:**
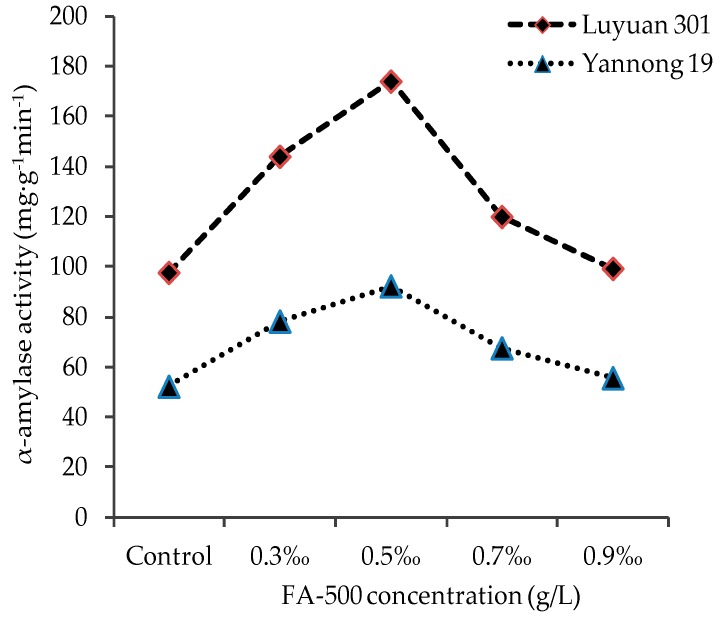
The effects of FA-500 with different concentrations on the activity of α-amylase.

**Figure 6:**
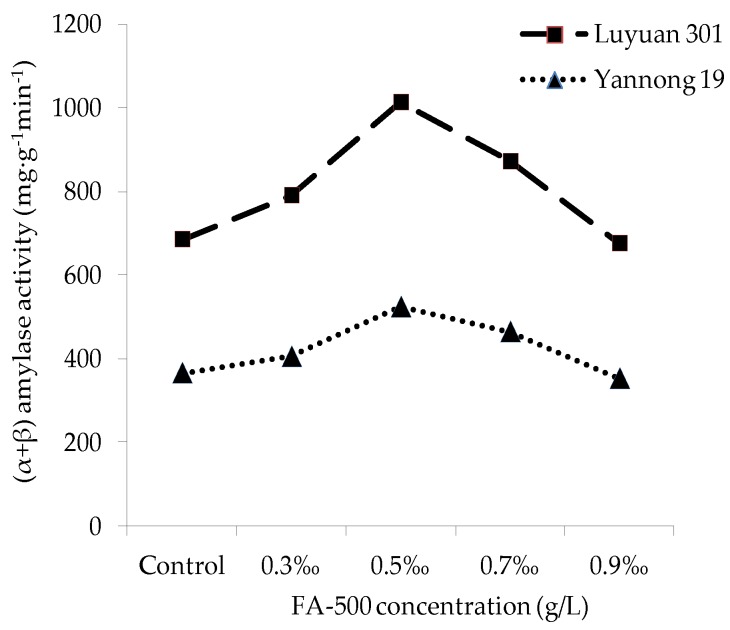
The effects of FA-500 with different concentrations on the activity of (α+β) amylase.

Figure 5 and Figure 6 illustrated that the effect of FA-500 with different concentration on the activity of α-amylase and (α+β) amylase was remarkable. In the groups of 0.3‰, 0.5‰ and 0.7‰, the activity of the two amylases was higher than the control group. When the concentration of FA-500 came up to 0.5‰, both had the most significant effect (*p* < 0.05). At the top, the activities of α-amylase and (α+β) amylase for Yannong 19 increased by 76.6% and 43.3% respectively, and 80.0% and 53.2% respectively for Luyuan 19. When the concentration was higher than 0.5‰, the two indexes both showed a downward trend with the increase of concentration. At the concentration of 0.9‰, the indexes began to be less than the control groups. In general, with an increase in FA-500 concentration, the increasing or decreasing amplitude of α-amylase activity was larger than (α+β) amylase for both kinds of seed. In the process of seed germination, the amylase can hydrolyze starch to monose. The improvement of amylase activity is beneficial to increase the concentration of the monose needed by respiration, which is helpful to enhance the respiration of seeds. The results revealed that FA-500 may affect the amylase activity in the process of seed germination in order to influence the growth of the seed.

## 3. Materials and Methods

### 3.1. Materials

Lignite was collected from Xiaopengzu coal mine in Eshan county of Yunnan province; then, its physical and chemical properties were analyzed, and the results are shown in Table 3. The selected wheat cultivars are Yannong 19 and Luyuan 301, which were examined and approved by Crop Variety Approval Committee of Shandong Province, PR China (Approval Nos. 2001001 and 2007044). The identification of the genotype refers to these papers [25,26,27]. Hoagland’s nutrient solution was prepared according to Hoagland et al. [28]. C_8_-C_40_ *n-*alkane mixed standard was bought from AccuStandard company of the U.S. Thirty percent hydrogen peroxide was purchased from East Tengen Reagent Factory of Tianjin. The MD31 dialysis bag was bought from Spectrum labs of the U.S. The Agilent GC/MS 6890N/5975C was bought from Agilent of the U.S. The R100 rotary evaporator was bought from Shanghai Shen Shun Biotechnology Co. Ltd. The freeze dryer was from Shanghai lang Instrument Co., Ltd. The centrifugal machine was from the Shanghai anting instrument plant.

### 3.2. Preparation of 500-FA

One liter of 30% (*v/v*) H_2_O_2_ was added in the reactor with 1 kg sieved brown coal, continuously stirred for 3 h at 40 °C. When the oxidation reaction was finished, the product was centrifuged for 15 min (speed 3500 r/min), and the filtered supernatant fluid was the degradation liquid. The degradation liquid was concentrated and dried by a vacuum-rotary evaporation procedure at 50 °C. Solid FA was dialyzed with a 100–500 DA bag, and then, the dialysis fluid was concentrated and freeze-dried into a solid. The 0.3‰, 0.5‰ and 0.7‰ FA-500 solutions were prepared with distilled water by mass:volume (g/L).

### 3.3. Chromatographic Conditions

Column: HP-FFAP fused silica capillary column (30.0 m × 0.25 mm × 0.25 μm); carrier gas: He; flow rate: 2.0 mL/min; constant current mode; injector temperature: 180 °C; split injection, split ratio of 10:1; the initial temperature was maintained at 60 °C for 7 min, programmed to 180 °C at a rate of 2 °C/min and held for 10 min; transmission line temperature: 280 °C.

#### 3.3.1. MS Conditions

Using an electron bombardment iron source under the condition of electronic energy of 70 eV; electron multiplier voltage: 980 V; solvent delay time: 7 min; the temperature of ionization source and quadrupole were set as: 250 °C, 150 °C; scanning mode: full scan, mass range of *m/z* 50–550 atomic mass units; and the scan rate was 0.5 scan/s.

#### 3.3.2. Measurement of RI

**Figure 7:**
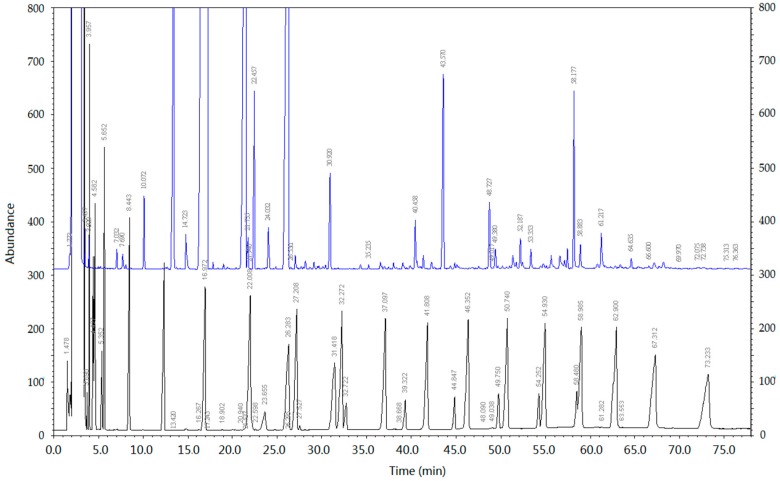
Gas chromatogram of FA-500 and C_8_-C_40_ *n*-alkanes. The top is the gas chromatogram of FA-500; the bottom is the gas chromatogram of C_8_-C_40_ *n*-alkanes.

According to the conditions mentioned above, each *n*-alkane standard peak retention time value was measured with a linear heating program of the retention index formula to calculate the components of the RI values:

RI = 100Z + 100 [TR (X) − TR (Z)]/[TR (Z + 1) − TR (Z)]
(1)

wherein: TR (X), TR (Z), TR (Z + 1) are the peak retention time value of the sample and *n*-alkanes standards substance whose carbon atoms were Z and Z + 1, respectively; moreover, TR (Z) < TR (X) < TR (Z + 1). The RI of the analyzed components was obtained through calculating the retention time of the components obtained by GC, to verify the structure of the component further [29,30]. The retention time of FA-500 and mixed standards (C_8_-C_40_ *n*-alkanes) is shown in Figure 7.

### 3.4. Seed Germination Process

The plump seeds were disinfected with 10% NaClO for 10 min and then washed 7 times with sterile distilled water. Using distilled water as the blank control and FA-500 solution with different concentrations as the sensitized liquid, the seeds were soaked in these respectively for two hours. One-hundred fifty grains of sensitized seeds from the same batch were put into three petri dishes with 50 grains per petri dish. The bottom of the petri dishes with a diameter of 12 cm was covered by a layer of filter paper, and 10 mL Hoagland’s nutrient solution were added into each petri dish. The seeds were cultured in a constant temperature incubator as 16–26 °C during the day and 10–16 °C at night, and the average photoperiod was 14 h. The evaporative loss of water was obtained through the weight method and compensated by adding distilled water into petri dishes. After 72 h, the germination rate, coleoptile and radicle length, germination index, vitality index and activity of α-amylase and (α+β) amylase were measured respectively according to the methods of Section 3.5.

### 3.5. Measurement of Activity Index

This was the standard of seed germination that the length of the seed radicle was more than the seed length by 1/2. After 72 h, the coleoptile and radicle length of 20 germinating seeds from each group were measured randomly, and the germination rate, germination index and vitality index were calculated according to the following formulas.

Germination rate (GR) = ∑G_t_/T × 100% (2)

Germination index (GI) = ∑G_t_/D_t_ (3)

Vitality index (VI) = S × GI (4)
(4)

G_t_ is the number of germinations on the t-th day; D_t_ is the germination number accordingly; T is the total number of seeds; S is the length of radicle. After 72 h, the activity of the seeds of α-amylase and (α+β) amylase was measured using 3, 5-2 nitro salicylic acid [31]. 

## 4. Conclusions

The chemical constituents of FA-500 and their molecular weight were analyzed and identified by using GC-MS combined with RI in this study. Moreover, the activity of FA-500 on promoting wheat seed germination was explored preliminarily. It could be concluded that FA-500 can produce a significant effect on promoting wheat seed germination in the appropriate concentration range, while it showed an inhibiting effect with a high concentration. In the process of seed germination, FA-500 may affect the growth of the seed through influencing the amylase activity, which was related to respiration. The results are consistent with the research of Zhang Hui et al. [18,32], and their study found that small molecules of FA showed a greater promoting activity.

In addition, fulvic acid was also found to overcome the rate-limiting step of transport of Fe from soil solution to plant roots by diffusion, and the efficiency of Fe-FA as a fertilizer is much greater than that of FeCl_3_ [8]. This may be related to the complexation of metal ions and oxygen-containing functional groups (such as carboxyl, phenolic hydroxyl, alcoholic hydroxyl and quinonyl group) belonging to FA [33]. Humic substances can enhance the availability of Zn and Fe, which were able to improve the activity of many enzymes [34]. The FA-500 with lower molecular weight has higher relative content of oxygen-containing functional groups, which probably produces more advantages for the diffusion and ion regulation in the plant. In addition, the activity characteristics of FA-500 were very similar to plant hormones with a promotive effect in this study. Many other studies also indicated that humic acids, particularly small molecule fractions, often show a hormone-like effect. Piccolo et al. found that humic acids had auxin-like, gibberellin-like and cytokinin-like activity, and the small molecule fractions could produce higher activity [17]. In the research of Nardi et al., the humic substances exhibited auxin-like and gibberellin-like activity [35]. Indoleacetic acid was directly detected in the humus by Muscolo et al. [36]. Meanwhile, the terpenes, phenols and quinones in humic acids or fulvic acids also have been confirmed to have indirect activities on promoting growth or resisting adversity [18,37,38]. Although plant hormone was not directly detected in the FA-500, it was not ruled out that some of these small molecules were the precursors or intermediates to form plant hormones or their analogues. Furthermore, the activity mechanism of F-500 may be similar to plant hormones and their analogues.

The study confirmed again that small molecule fraction of fulvic acids had strong activities at very low concentrations. FA-500, as a mixture consisting of small molecule organic compounds with a high activity, is very suitable for the development of high efficiency organic fertilizer or additives such as foliar fertilizer, drip irrigation fertilizer and plant growth regulators. This implies potential large economic value. The component identification and activity investigation of FA-500 in this study may provide a theoretical basis and technical support for the development of the high-end HA fertilizer. The focuses of future research on FA will be the activity of a single component, the interaction among different components and the molecular mechanism of activity. 

## Figures and Tables

**Table 1:** Chemical constituents of the FA-500 analyzed by GC-MS. RI, retention index.

NO.	Compound	Retention Time (min)	Molecular Formula	Similarity (%)	RI	Area Percentage (%)
1	1,5-Hexadiene-3-ol	9.633	C_6_H_10_O	85.9	1032	1.425
2	Ethyl glycolate	10.545	C_4_H_8_O_3_	98.7	1055	3.927
3	Acetic acid	11.694	C_2_H_4_O_2_	93.9	1084	0.104
4	Methoxyacetic acid	11.739	C_3_H_6_O_3_	92.6	1085	0.121
5	Ethyl methyl ether	11.796	C_3_H_8_O	95.6	1087	0.487
6	Propan-1-ol	11.825	C_3_H_8_O	96.8	1088	0.157
7	Propanedioic acid, oxo-, ethyl methyl ester	11.870	C_6_H_8_O_5_	96.0	1091	0.224
8	Diethyl oxalate	14.388	C_6_H_10_O_4_	98.3	1144	6.745
9	Propanedioic acid, methyl, ethyl ester	16.509	C_6_H_10_O_4_	92.6	1190	0.394
10	Diethyl malonate	18.655	C_7_H_12_O_4_	96.3	1233	17.156
11	Glucose	18.784	C_6_H_12_O_6_	74.8	1236	0.323
12	Ethyl levulinate	19.520	C_7_H_12_O_3_	91.8	1251	2.117
13	Butanedioic acid, ethyl methyl ester	20.897	C_7_H_12_O_4_	94.9	1278	0.532
14	Diethyl methylsuccinate	21.188	C_9_H_16_O_4_	93.7	1284	0.666
15	Diethyl succinate	23.452	C_8_H_14_O_4_	92.7	1334	29.512
16	Ethyl 4-acetylbutyrate	25.256	C_8_H_14_O_3_	97.1	1376	0.119
17	Diethyl glutarate	28.145	C_9_H_16_O_4_	82.5	1522	2.235
18	Hexanedioic acid diethyl ester	33.825	C_10_H_18_O_4_	82.2	1732	0.202
19	Ethyl 3-hydroxy-4-methylpentanoate	36.317	C_8_H_16_O_3_	91.8	1784	0.133
20	3-(methylthio)-propionaldehyde	37.628	C_4_H_8_OS	77.9	1811	0.487
21	Maleic acid diethyl ester	40.808	C_8_H_14_O_5_	98.0	1879	2.658
22	Succinic acid, 2-hydroxy-3-methyl-, diethyl ester	46.054	C_9_H_16_O_5_	86.3	1993	0.829
23	Diethyl 3-hydroxyglutarate	46.762	C_9_H_16_O_5_	91.7	2009	0.159
24	Diethyl 2-acetylglutarate	49.274	C_11_H_18_O_5_	83.6	2067	0.331
25	Ethyl 2-ethylacetoacetate	49.781	C_8_H_14_O_3_	83.0	2078	0.170
26	Ethyl oxamate	49.987	C_4_H_7_NO_3_	87.5	2083	0.231
27	2-(1-Ethoxyethoxy)-3-methysuccinic acid, diethyl ester	51.244	C_13_H_24_O_6_	87.8	2112	0.397
28	6-desoxy-l-gulitol	51.303	C_6_H_14_O_5_	80.1	2115	1.282
29	d-glucosiduronic acid	52.522	C_6_H_10_O_7_	80.1	2143	0.226
30	Levulinic acid	52.995	C_5_H_8_O_3_	86.4	2154	0.470
31	d-manno-2-Heptulose	53.376	C_7_H_14_O_7_	81.4	2163	0.249
32	Ethyl hydrogen malonate	54.294	C_5_H_8_O_4_	98.1	2185	5.070
33	Tetraethylene glycol di-2-ethylhexoate	54.528	C_24_H_46_O_7_	83.0	2190	0.156
34	Ethyl(2-tetrahydropyranyl)acetate	54.702	C_9_H_16_O_3_	78.7	2195	0.230
35	Ethyl hydrogen succinate	55.784	C_6_H_10_O_4_	96.0	2221	11.388
36	Diethyl 4-oxopimelate	56.370	C_11_H_18_O_5_	81.5	2236	0.846
37	Succinic acid imide	58.522	C_4_H_5_NO_2_	84.2	2288	0.412
38	4-Dihexylcarbamoyl-butyric acid	58.819	C_17_H_33_NO_3_	81.4	2296	0.581
39	Hexanedioic acid, 3-oxo-, diethyl ester	60.846	C_10_H_16_O_5_	86.4	2347	0.518
40	Monoethyl itaconate	61.055	C_7_H_10_O_4_	87.7	2353	0.272
41	Diethyl 2-aminomalonate	62.080	C_7_H_13_NO_4_	84.7	2379	0.277
42	dl-glutamine	64.203	C_5_H_10_N_2_O_3_	81.4	2430	0.233
43	2-Ethyl-3-formylaminosuccinic acid, di-t-butyl ester	65.068	C_15_H_27_NO_5_	83.7	2449	0.235
44	Isobutyl 3-(perhydro-5-oxo-2-furyl)propionate	65.568	C_11_H_18_O_4_	78.5	2461	0.216
45	Diethyl allylmalonate	68.679	C_10_H_16_O_4_	81.8	2523	0.146
46	Cis-9,10-Epoxyoctadecanamide	74.336	C_18_H_35_N_2_O_2_	82.2	2615	0.128
47	Dodecanoic acid, 2-(2-hydroxyethoxy)ethyl ester	74.975	C_16_H_32_O_4_	81.9	2623	0.174

**Table 2:** The effects of FA-500 with different concentrations on wheat seed germination.

Treatment	Germination Rate/(%)		Length of Coleoptile/mm		Length of Radicle/mm	
	Yannong 19	Luyuan 301	Yannong 19	Luyuan 301	Yannong 19	Luyuan 301
Control	91.5 ± 0.3	78.0 ± 1.2	29.3 ± 0.3	28.9 ± 0.4	36.4 ± 0.2	34.1 ± 0.3
0.3‰	92.0 ± 0.4	76.0 ± 0.6 *	29.7 ± 0.1	30.0 ± 0.2	35.1 ± 2.0	35.0 ± 0.4
0.5‰	92.5 ± 0.8	83.0 ± 2.5	32.0 ± 0.4	30.7 ± 0.7 *	41.9 ± 0.6 *	39.5 ± 0.7 *
0.7‰	82.5 ± 2.9 *	78.8 ± 0.8	30.2 ± 0.8	29.2 ± 0.6	40.6 ± 1.4	38.3 ± 0.4 *
0.9‰	83.0 ± 1.2 *	77.5 ± 1.5	36.1 ± 1.2 *	27.6 ± 0.4	36.1 ± 0.4	35.4 ± 0.5

Results are expressed as the mean ± S.D. (* *p* < 0.05 vs. the control).

**Table 3:** The main physicochemical properties of the raw coal. HA, humic acid; FA, fulvic acid.

Sample	The Water Content (%)	The Ash Content (%)	The Total HA Content (%)	The Free HA Content (%)	The FA Content (%)
**Eshan brown coal**	18.30	13.16	51.29	53.25	1.07
